# Supplementary material for: Luminosity thresholds of colored surfaces are determined by their upper-limit luminances empirically internalized in the visual system
Source: J Vis. Author manuscript; Available in PMC 2022 Jan 4. (PMC8662570; doi:10.1167/jov.21.13.3)
Supplement: Appendix [file EMS140546-supplement-Appendix.pdf]

Appendix

Individual observer settings in Experiment 2

In the main text, we presented mean observer settings for Experiment 2. Figure A1 shows the individual observer settings. There is some individual variation, but, overall, the trend was similar across individuals.

Implementation of other past models

Here, we compare two simplistic models that predict luminosity thresholds based on post-receptoral signals or cone signals of the test field. Note that these two models were also implemented as candidate models in Speigle & Brainard (1996).

The first model is a post-receptoral model which assumes that the visual system monitors the weighted sum of the three types of cone signals of the test field. The test field appears self-luminous when $w_L L_T + w_M M_T + w_S S_T >= 1$, where L_T, M_T and S_T denote cone signals of the test field and w_L, w_M, and w_S denote weightings for each class of cone signal ($w_L >= 0$, $w_M >= 0$, and $w_S >= 0$). Then, we optimized weightings w_L, w_M, and w_S, which produced the minimum root mean square error between model prediction and mean observers' setting.

The second model is a generalized Evans's model (Evans, 1959) which assumes that a test field appears self-luminous when any of L_T, M_T, or S_T cone signals exceed a certain criterion level. Thus, the model predicts that a test field is self-luminous when $L_T >= c_L$, $M_T >= c_M$ or $S_T >= c_S$. The goal of the optimization here was to find criterion c_L, c_M, and c_S that minimized the root mean square error between model prediction and mean observers' setting.

For both models, the optimization procedure was performed separately for each experimental condition. In other words, for example, in Experiment 2, we performed optimization procedures nine times in total (3 illuminants × 3 distributions).

Figures A2, A3, and A4 depict the prediction of the post-receptoral model and the generalized Evans's model as well as the optimal color model in experiments 1, 2, and 3. To obtain the prediction of the optimal color model, we used the ground-truth illuminant for experiments 1 and 3, but for Experiment 2, we used the estimated illuminant so that the peak is matched between the prediction of optimal color model and the mean observer settings (for more details, see the Results section in Experiment 2 in the main text).

For Experiment 1, we see that the post-receptoral model predicts a linear luminosity threshold locus over L/(L + M) which did not lead to a high correlation coefficient (shown at the right upper corner in each panel). The generalized Evans's model came closer to observer settings, but in all conditions the correlation coefficient was lower than that of the optimal color model. Welch's *t*-test (one-tailed, no assumption about equal variance) on averaged correlation coefficients across the five conditions showed that the optimal color model has a significantly higher correlation than the Evans' model ($t(4.64) = 4.57$, $p = 0.0072$).

Downloaded from jov.arvojournals.org on 12/09/2021

A quite similar trend is shown in Experiment 2, although in one condition (flat and 20,000 K), the Evans's model exceeded the optimal color model's correlation coefficient. However, again Welch's t-test on averaged correlation coefficients across the nine conditions showed a significantly higher correlation coefficient for the optimal color model than the Evans's model ($t(8.95) = 3.72$, $p = 0.0048$).

In contrast, for Experiment 3, we see that the Evans's model showed higher correlations than the optimal color model especially in green illuminant conditions. Welch's t-test on averaged correlation coefficients across the 12 conditions showed that there is no significant difference between the Evans's model and the optimal color model ($t(12.2) = 1.80$, $p = 0.0962$).

In summary, it is evident that for experiments 1 and 2, the optimal color model predicts human observer settings better than the two alternative models considered here. In Experiment 3, there was no significant difference in correlation coefficients between the Evans's model and the optimal color model. One interpretation would be that when the scene illuminant is atypical, human observers rely on the simple statistics such as cone signals because the visual system does not know the optimal color locus under the atypical illuminant.

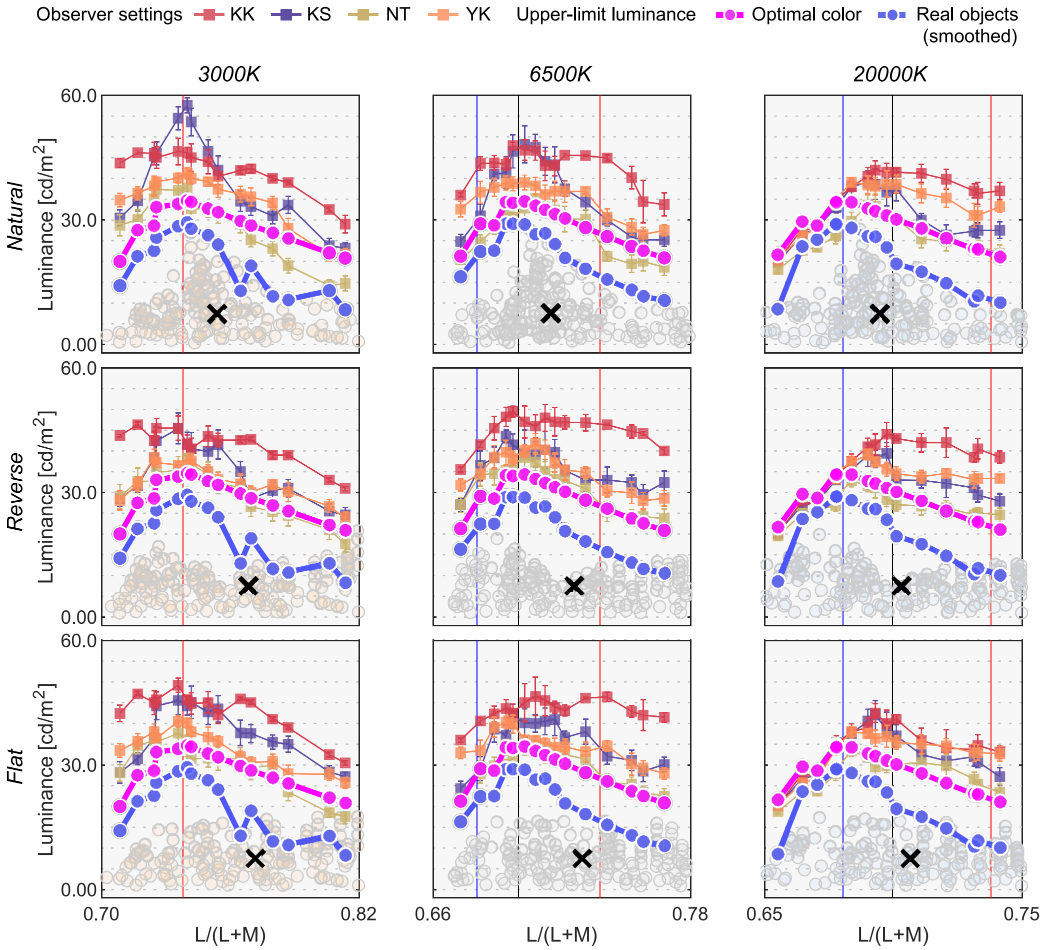

Figure A1. Individual observer settings in Experiment 2. Colored square symbols indicate the averaged setting across 10 repetitions for each observer. The error bar indicates ±1 SE across 10 repetitions. The magenta circles denote the optimal color locus and the blue circles show the real object locus. The red, black, and blue vertical solid lines show the chromaticities of the 3000 K, 6500 K, and 20,000 K test illuminants, respectively. The black cross symbol indicates mean LMS value across surrounding stimuli. Note that the horizontal range differs across panels.

Downloaded from jov.arvojournals.org on 12/09/2021

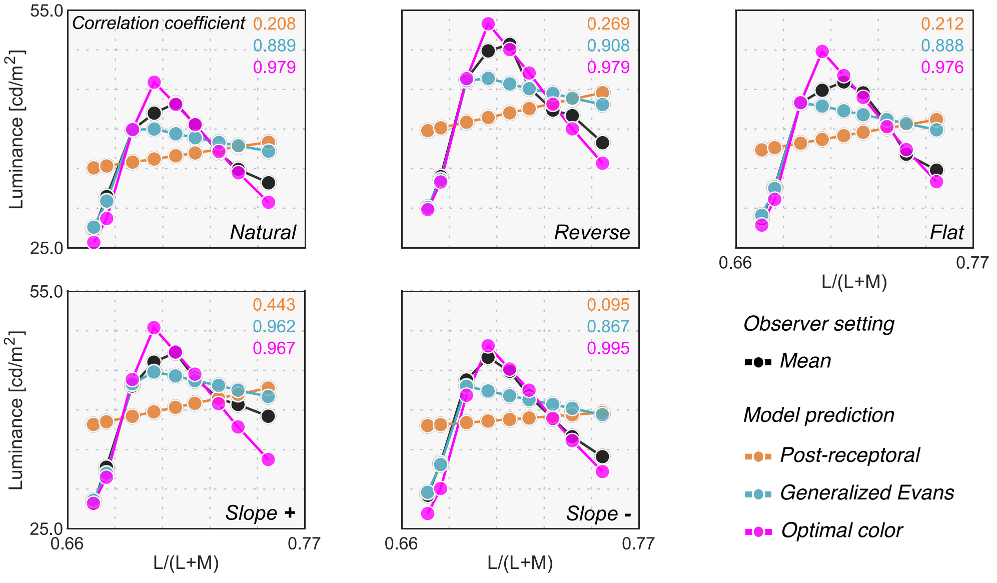

Figure A2. Predictions from the post-receptoral model, the generalized Evans's model and the optimal color model in Experiment 1. Each model prediction was scaled to give the minimum root mean square error between model prediction and mean observer setting to compare their shapes more easily. The correlation coefficients between model prediction and mean observer settings are shown at the top right corner in each panel (in the order of post-receptoral model, generalized Evans's model and optimal color model from top to bottom). We used a ground-truth illuminant (i.e. 6500 K) to obtain the prediction from the optimal color model.

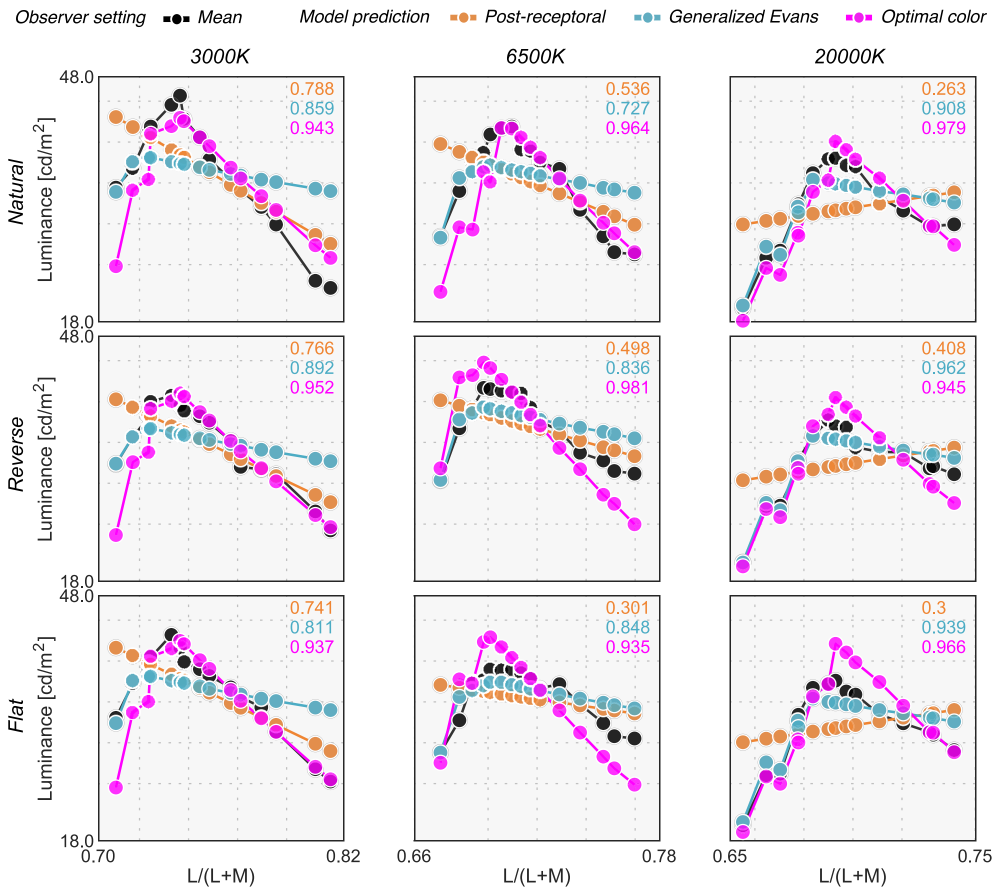

Figure A3. Predictions from the post-receptoral model, the generalized Evans's model, and the optimal color model in Experiment 2. Each model prediction was scaled to give the minimum root mean square error between model prediction and mean observer setting. The correlation coefficients between model prediction and mean observer setting are shown at the right top corner in each panel. For the optimal color model, we used an estimated illuminant whose peak matched that of the observer settings (see Results section in Experiment 2 in the main text for more details).

Downloaded from jov.arvojournals.org on 12/09/2021

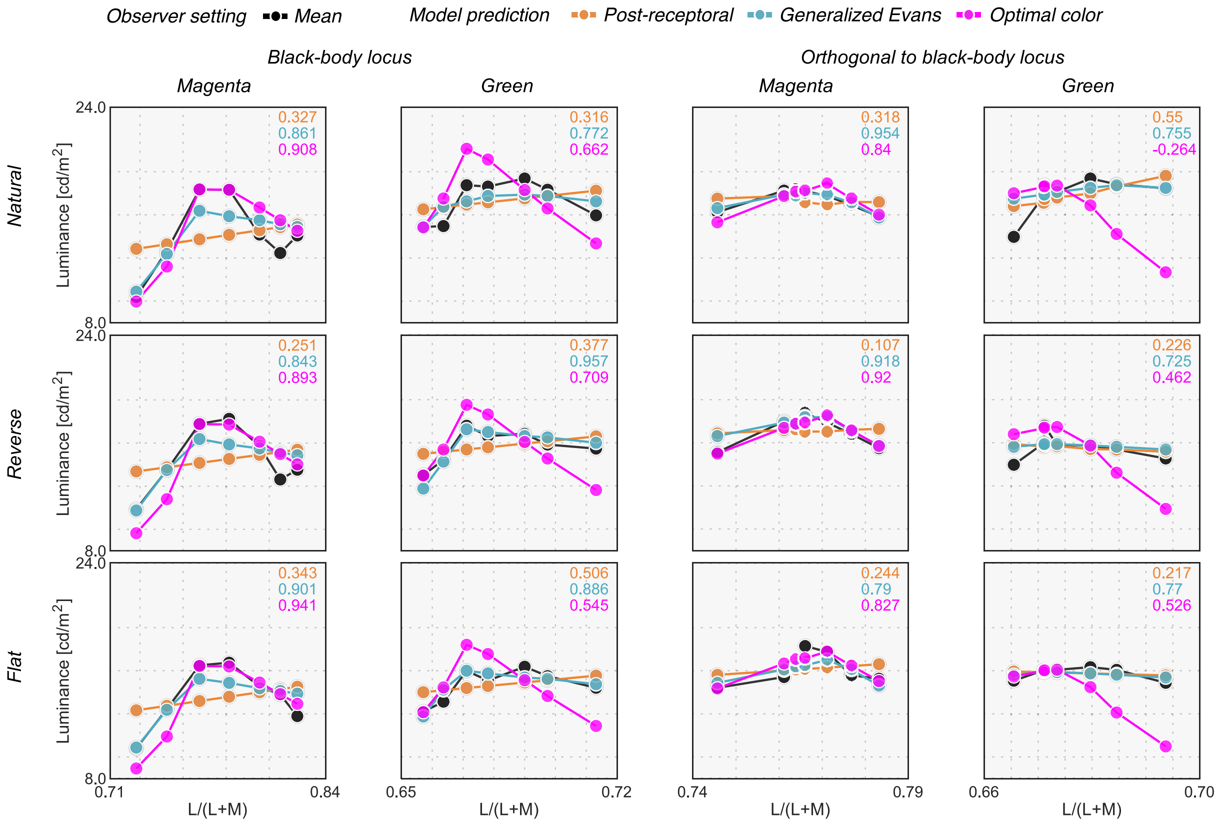

Figure A4. Predictions from the post-receptoral model, the generalized Evans's model, and the optimal color model in Experiment 3. Each model prediction was scaled to give the minimum root mean square error between model prediction and mean observer setting. The correlation coefficient between model prediction and mean observer setting is shown at the top right corner in each panel. We used a ground-truth illuminant (i.e. magenta or green) for the optimal color model.

Downloaded from jov.arvojournals.org on 12/09/2021
